# Knowledge and Perception of the Population of the Hail Region Toward Short Stature in Children

**DOI:** 10.7759/cureus.87176

**Published:** 2025-07-02

**Authors:** Reem A Alshammari, Walaa A Alshangity, Rana N Almansour

**Affiliations:** 1 Paediatrics, University of Hail College of Medicine, Hail, SAU; 2 College of Medicine, University of Hail College of Medicine, Hail, SAU

**Keywords:** children, hail, kingdom of saudi arabia (ksa), knowledge and perception, short stature

## Abstract

Background: Short stature is defined as height that is more than two standard deviations (SDs) below the corresponding mean height for a given age, sex, and population. It can be a normal variant or result from pathological conditions, such as genetic disorders, endocrine abnormalities, or chronic diseases. Early diagnosis and timely management are crucial for effectively addressing this condition.

Aim: This study assessed the knowledge and awareness of the population of Hail, Saudi Arabia regarding pediatric short stature, its detection, and possible interventions to correct it.

Subject and methods: This cross-sectional study was conducted in the Hail region, Saudi Arabia. An online questionnaire was designed and distributed through various social media applications. It includes informed consent of participants, sociodemographic data, and questions about short stature causes, detection, and intervention and attitudes towards short stature in children. Data analysis was performed using IBM SPSS Statistics for Windows, Version 26 (Released 2019; IBM Corp., Armonk, New York, United States).

Results: We received 864 responses, and the majority of participants (74.8%) had poor knowledge regarding short stature in children, while only 25.2% demonstrated good knowledge.

Conclusion: In this study, the majority of participants demonstrated poor knowledge regarding pediatric short stature, with only 25.2% achieving a good knowledge score. Higher knowledge levels were significantly associated with younger age, higher education, being unmarried, and not having children. These findings highlight the need for targeted educational strategies to improve awareness, early recognition, and timely healthcare-seeking behavior.

## Introduction

Short stature is a common concern among parents and is the leading cause of referral to pediatric endocrinologists [1]. Although referral guidelines differ greatly across countries, early diagnosis and timely treatment are crucial, especially in children who require growth hormone therapy [2]. Short stature is defined as a height below average compared to that of children of the same sex and age, while also considering family height. It is typically diagnosed using the 3rd percentile on growth charts in pediatric clinics as the demarcation of the lower limit. A child whose height falls two or more standard deviations (SDs) below the mean for children of the same sex and chronological age is considered to have short stature [3]. Most children referred for short stature are ultimately found to have normal growth variants, such as familial short stature or constitutional delay of growth and puberty. However, a minority of these cases are caused by an underlying pathology. Pathological causes include endocrine disorders such as growth hormone deficiency and hypothyroidism, chronic diseases such as cardiac, renal, or hepatic conditions, genetic syndromes such as Turner syndrome and Noonan syndrome, metabolic diseases, and other rare conditions. In some cases, short stature can be an initial sign of any of these underlying pathologies [4,5]. The evaluation of short stature involves serial growth monitoring, as growth failure is defined as a deflection of the growth curve. Further assessment is required for children with heights below the third percentile on growth charts tailored to age, sex, and ethnicity. World Health Organization (WHO) growth charts are suitable for children up to two years of age, whereas local growth charts are recommended for older children. Understanding the etiology behind short stature is essential for identifying treatable causes and ensuring optimal growth for affected children [6]. 

In Saudi Arabia, a national reference study involving 19,372 healthy children and adolescents aged 5 to 17 years reported that the prevalence of moderate short stature (height-for-age < -2 SD) was 11.3% in boys and 10.5% in girls, while the prevalence of severe short stature (< -3 SD) was 1.8% and 1.2%, respectively [7]. These figures highlight that short stature affects a substantial proportion of Saudi children, emphasizing the need for public awareness and early medical evaluation. 

Parental awareness plays a critical role in facilitating timely consultation and treatment adherence. Despite the clinical and public health significance of this issue, no previous studies have assessed the knowledge and perception of short stature among residents of the Hail region. Therefore, the present study aims to evaluate the awareness and attitudes of the local population in Hail, Saudi Arabia, towards pediatric short stature. Enhancing public understanding in this area may support early recognition, appropriate referrals, and improved long-term outcomes for affected children.

## Materials and methods

This cross-sectional study included a total of 864 participants. The estimated sample size was calculated to be 384 using the Raosoft sample size calculator, based on a 95% confidence level and a 5% margin of error. A non-probability convenience sampling technique was used in this study; the questionnaire was distributed online via various social media platforms across the Hail region, Saudi Arabia, during the period from September to November 2024. 

The study has been reviewed and approved by the Research Ethics Committee (REC) at the University of Hail, with the research number H-2024-455 and dated 7/10/2024. Participants were informed that their participation was voluntary and that all responses would be kept anonymous and confidential. No personal identifiers were collected. All authors declare that informed consent was obtained from all participants for publication of the original article.

The study goals were clarified at the beginning of the questionnaire, thus enabling the participants to fully understand the aims of the study and make an informed decision whether they wished to participate in the study or not. 

The questionnaire used in this study was adapted from a previously published survey that assessed public knowledge of pediatric short stature [8]. Additional modifications were made to suit the objectives of the present study, with input and guidance from a consultant pediatrician. 

The questionnaire measured several variables and was composed of two sections. The first section covered participants’ sociodemographic characteristics, including age, sex, nationality, residence, educational level, marital status, and job sector. The second section included questions about the definition of pediatrics short stature, its causes, detection, and possible interventions. Potential participants comprised both Saudi and non-Saudi men and women, aged 18 years or older, living within the Hail region, Saudi Arabia.

The data analysis for this study was carried out using IBM SPSS Statistics for Windows, Version 26 (Released 2019; IBM Corp., Armonk, New York, United States). The overall knowledge score was calculated based on six knowledge-based questions addressing the definition, causes, timing of medical consultation, whether short stature can be a normal form of growth, healthcare professionals involved, and treatment options for pediatric short stature. Each correct answer was given one point, and participants were classified based on a 60% cut-off: those who scored below 60% were considered to have poor knowledge, whereas those who scored 60% or higher were considered to have good knowledge. Descriptive statistics were used to summarize the participants’ demographic information, including frequencies and percentages for categorical variables such as age, sex, and education level, and the mean and SD for continuous variables such as age. To assess the factors associated with knowledge level, chi-square tests were conducted to compare different groups (such as age, sex, marital status, and education) and their knowledge levels (poor vs good). Statistical significance was set at p < 0.05.

## Results

This study included 864 participants from the Hail region of Saudi Arabia. Table 1 presents the sociodemographic characteristics of the study participants. The age distribution indicated that the majority of participants were between 18 and 35 years of age, with the largest group aged 18-25 years (35.2%). The mean age of participants was 34.6 years (SD = 11.4). Female participants constituted 71.9% of the participants, and male participants accounted for 28.1%. Most participants (96.8%) were Saudi nationals, while a small percentage (3.2%) were non-Saudi nationals. In terms of residence, 81% lived in Hail city, whereas the remaining 19% resided in nearby villages. The educational level of the participants was generally high, with 68.4% having completed a university education. Regarding marital status, the majority were married (58%), followed by singles (38.1%). Employment data showed that nearly half of the participants were unemployed (45.1%), and 40.5% worked in non-healthcare sectors. The study also revealed that more than half the participants (57.8%) had children, with a smaller group (4.4%) fostering children who were not their own. 

**Table 1:** Sociodemographic characteristics of the study participants from the Hail region, Saudi Arabia (n=864)

Personal Data	No.	%
Age in years		
18-25	304	35.2%
26-35	172	19.9%
36-45	190	22.0%
46-55	144	16.7%
> 55	54	6.3%
Sex		
Male	243	28.1%
Female	621	71.9%
Nationality		
Saudi	836	96.8%
Non-Saudi	28	3.2%
Place of Residence		
Hail	700	81.0%
Villages near Hail	164	19.0%
Educational level		
Below secondary	22	2.5%
Secondary	196	22.7%
University	591	68.4%
Post-graduate	55	6.4%
Marital status		
Single	329	38.1%
Married	501	58.0%
Divorced/widowed	34	3.9%
Job Sector		
Not working	390	45.1%
Student	28	3.2%
Non-healthcare field	350	40.5%
Healthcare field	75	8.7%
Retired	21	2.4%
Do you have children?		
Yes	499	57.8%
No	327	37.8%
I foster children who are not my own	38	4.4%

Table 2 presents data about the family history of short stature and the timing of medical intervention among participants in the Hail region. Of the 864 participants, 33.1% reported a family history of short stature, while the majority (66.9%) did not. Among those with a family history, a significant proportion (52.1%) did not seek medical intervention at all, while 35.7% did so before puberty and only 12.2% after puberty. 

**Table 2:** Family history of short stature and timing of medical intervention in the Hail region population (n=864)

Variable	No.	%
Family history of short stature		
Yes	286	33.1%
No	578	66.9%
If yes, when was medical intervention initiated? (n=286)		
Before puberty	102	35.7%
After puberty	35	12.2%
They did not seek any medical intervention	149	52.1%

With regard to the knowledge of the population of Hail region of Saudi Arabia regarding short stature in children (Table 3), most participants (68.2%) defined short stature based on physical observations, such as noticing a child’s height compared to that of their siblings or peers, whereas a smaller proportion (26.4%) understood it in terms of growth curve standards. Most participants (87.5%) correctly identified familial (hereditary) short stature, with hormonal causes reported by 56.3%. Other causes such as malnutrition (46.4%) and staying up late (29.6%) were also reported, but there was less awareness of genetic diseases (syndromes) (27.8%) and constitutional delay (16.8%). A large majority (82.5%) knew that a doctor should be consulted before puberty if short stature was noticed. When asked whether short stature could be considered as a normal growth variation, 61.1% agreed that it could be in some cases. Most participants (65.4%) were aware that a pediatric endocrinologist should be consulted for short stature, and many (60.3%) said that therapeutic interventions, such as hormone replacement therapy, are a potential treatment. However, only a small percentage (1.2%) considered surgical intervention as a solution. 

**Table 3:** Knowledge of the population of Hail region, Kingdom of Saudi Arabia toward short stature in children (n=864)

Knowledge items	No.	%
Definition of short stature in children		
When you notice that the child is short compared with his siblings or peers of the same age and gender	589	68.2%
When the child’s height is below two standard deviations or less than 3% on the growth curves	228	26.4%
Delay in changing the child’s clothing sizes and continuing to wear them for a longer time	356	41.2%
Causes of short stature in children		
Familial (hereditary short stature)	756	87.5%
Hormonal causes (growth hormone deficiency or hypothyroidism)	486	56.3%
Malnutrition	401	46.4%
Staying up late at night	256	29.6%
Genetic diseases (syndromes)	240	27.8%
Constitutional delay	145	16.8%
Immunodeficiency diseases	106	12.3%
Chronic diseases (heart diseases, liver diseases, kidney diseases, malabsorption, asthma)	106	12.3%
Psychological causes	53	6.1%
Others	5	.6%
When do you think you should visit a doctor if you notice that your child has short stature?		
Before puberty	713	82.5%
After puberty	151	17.5%
Can short stature be considered a normal form of growth disorders?		
Yes, in some cases	528	61.1%
Never	151	17.5%
I do not know	185	21.4%
Which healthcare professionals should be consulted if a child has short stature?		
Pediatric Endocrinologist	565	65.4%
General Pediatrician	559	64.7%
Internal Medicine	74	8.6%
Orthopedics	13	1.5%
Others	9	1.0%
Type of interventions that can be used to treat short stature		
Therapeutic interventions (hormone replacement therapy)	521	60.3%
Surgical intervention	10	1.2%
Both of them	333	38.5%

**Figure 1:**
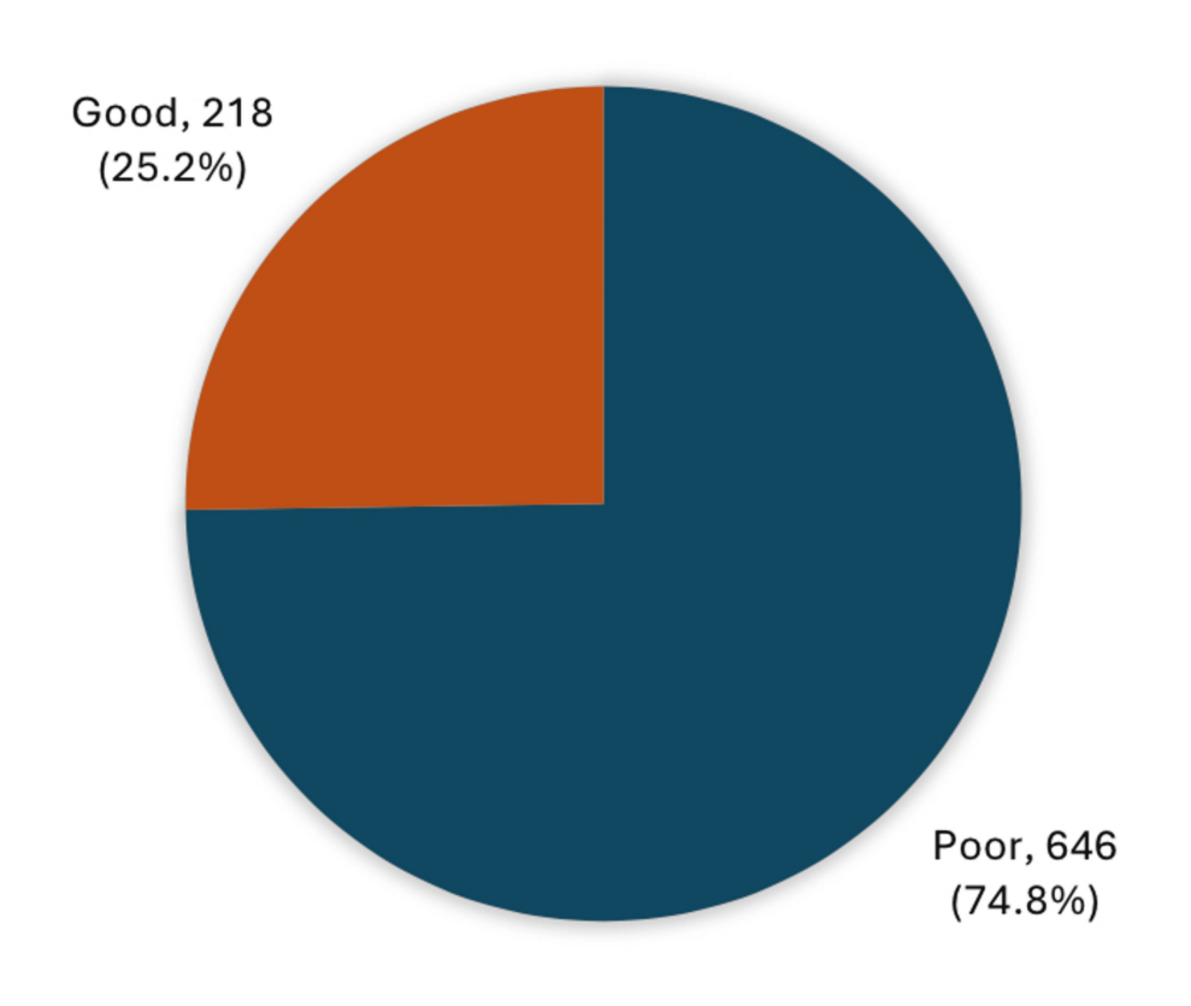
Overall knowledge among the population of Hail region, Kingdom of Saudi Arabia toward short stature in children (n=864)

Figure 1 highlights the overall knowledge level of the participants regarding short stature. A significant majority (74.8%) had poor knowledge about their condition, whereas only 25.2% had good knowledge. 

**Figure 2:**
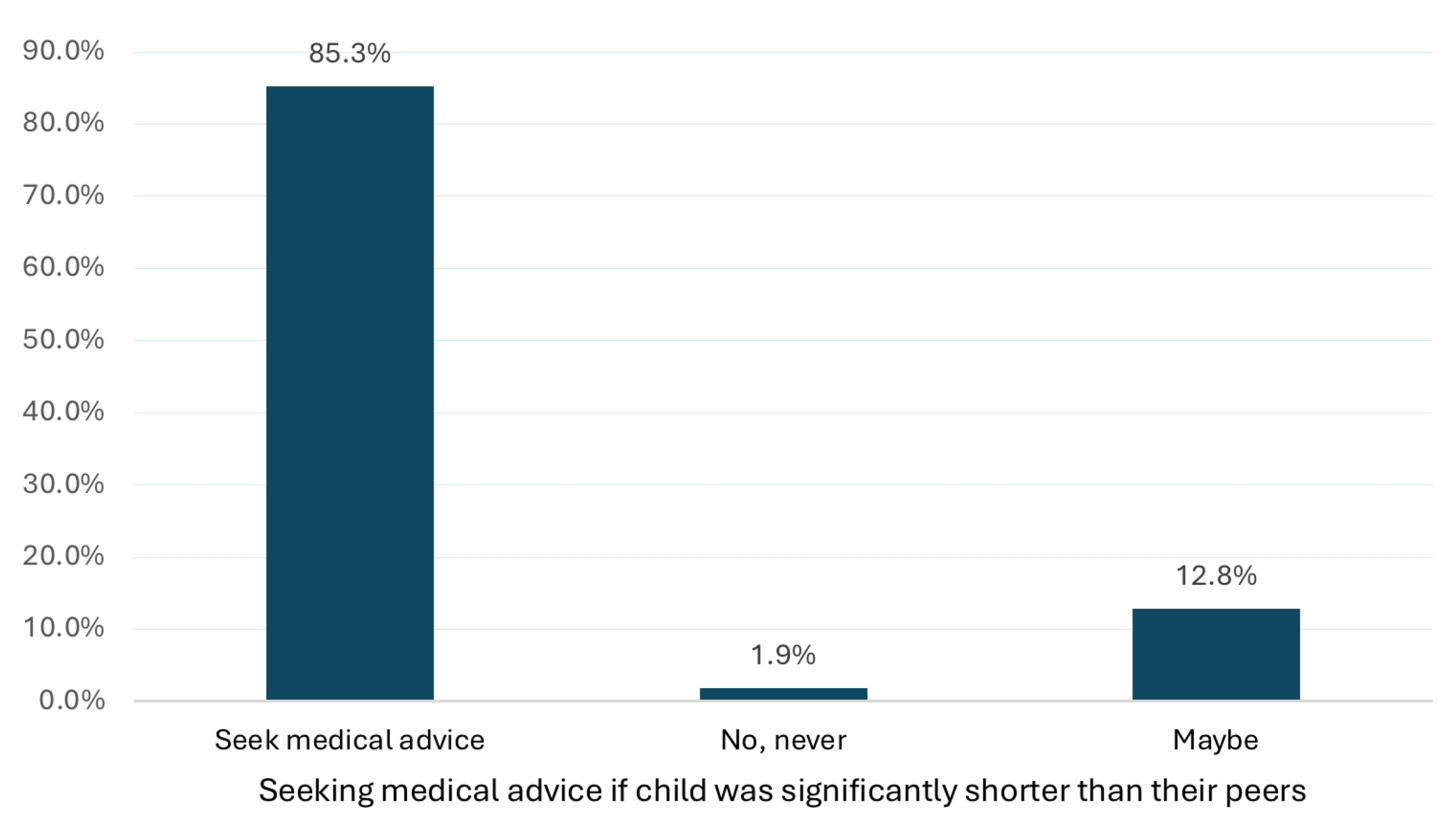
Attitudes toward seeking medical advice for short stature in children

Figure 2 illustrates the participants' attitudes towards seeking medical advice if their children were significantly shorter than their peers. Most participants (85.3%) reported that they would seek medical advice in such situations, whereas only a small percentage (1.9%) stated that they would never do so. Additionally, 12.8% were uncertain, answering "maybe”. 

Table 4 presents factors associated with the overall knowledge level of short stature among the population of Hail, Saudi Arabia. A significant relationship was observed between knowledge level and age. Younger individuals (18-25 years) had a relatively lower proportion of poor knowledge (60.2%) compared with older age groups, among whom poor knowledge increased significantly, especially in those aged 46-55 (85.4%) and over 55 years (87.0%) (p = 0.001). The educational level also significantly impacted knowledge. Participants with below secondary education had no good knowledge (0%), while those with secondary or university education exhibited better awareness, with 24.9% of university graduates categorized as having good knowledge (p = 0.033). Marital status was another influential factor. Single participants exhibited a significantly higher level of good knowledge (40.1%) compared with married individuals (15.8%) (p = 0.001). The job sector was another significant factor, as individuals working in the healthcare sector showed the highest percentage of good knowledge (26.7%), followed by those working in non-healthcare sectors (17.1%) (p = 0.001). Parental status showed a notable difference, with individuals without children demonstrating better knowledge. Among non-parents, 36.1% were classified as having good knowledge, compared with only 15.0% of parents (p = 0.001). There was no significant difference in knowledge based on family history of short stature (p = 0.063). Gender, nationality, and place of residence were not significantly associated with the knowledge level (p = 0.148, 0.392, and 0.901, respectively). 

**Table 4:** Factors associated with public knowledge about short stature in Hail, Saudi Arabia P: Pearson X^2^ test; ^: Exact probability test; *P < 0.05 (significant)

Factors	Overall knowledge level				p-value
	Poor		Good		
	No	%	No	%	
Age in years					
18-25	183	60.2%	121	39.8%	.001*
26-35	135	78.5%	37	21.5%	
36-45	158	83.2%	32	16.8%	
46-55	123	85.4%	21	14.6%	
> 55	47	87.0%	7	13.0%	
Sex					
Male	190	78.2%	53	21.8%	.148
Female	456	73.4%	165	26.6%	
Nationality					
Saudi	627	75.0%	209	25.0%	.392
Non-Saudi	19	67.9%	9	32.1%	
Place of Residence					
Hail	524	74.9%	176	25.1%	.901
Villages near Hail	122	74.4%	42	25.6%	
Educational level					
Below secondary	22	100.0%	0	0.0%	.033*^
Secondary	140	71.4%	56	28.6%	
University	444	75.1%	147	24.9%	
Post-graduate	40	72.7%	15	27.3%	
Marital status					
Single	197	59.9%	132	40.1%	.001*^
Married	422	84.2%	79	15.8%	
Divorced/widowed	27	79.4%	7	20.6%	
Job Sector					
Not working	270	69.2%	120	30.8%	.001*^
Student	16	57.1%	12	42.9%	
Non-healthcare field	290	82.9%	60	17.1%	
Healthcare field	55	73.3%	20	26.7%	
Retired	15	71.4%	6	28.6%	
Do you have children?					
Yes	424	85.0%	75	15.0%	.001*
No	209	63.9%	118	36.1%	
I foster children who are not my own	13	34.2%	25	65.8%	
Family history of short stature					
Yes	225	78.7%	61	21.3%	.063
No	421	72.8%	157	27.2%	

## Discussion

Pediatric short stature is an important issue requiring timely intervention whenever possible. Short stature is defined as a height that falls below two SDs from the mean for the child’s sex and chronological age [3]. This study aimed to explore the knowledge and perception of the population of the Hail region toward short stature in children.

Our study showed that only a small percentage of the population (26.4%) correctly defined short stature using growth curve standards, while the majority (68.2%) defined short stature based on physical observations, such as comparing a child’s height with that of siblings or peers. This likely reflects a reliance on visual or social cues rather than clinical tools, possibly due to limited awareness or limited use of standardized growth charts in routine child monitoring.

This finding aligns closely with another study conducted by a medical team at the College of Medicine, Qassim University [9], which assessed parents’ knowledge and perceptions of short stature across various regions in Saudi Arabia. In that study, 51.4% of parents were unsure whether a child was considered to have short stature if their height was 3-5% shorter than that of peers of the same age and sex, and 67.9% were unfamiliar with the Saudi growth chart. 

Both studies reveal a common tendency to rely on subjective comparisons rather than standardized growth assessments.

These findings may suggest a common misconception among the studied population that short stature can be diagnosed by observation alone, rather than using objective growth charts and clinical evaluation. This emphasizes the need for educational interventions to enhance parents’ understanding of short stature diagnostic criteria. Promoting the use of growth charts and encouraging regular pediatric evaluations may help address these gaps and improve early detection and management of growth-related issues in children.

Regarding the causes of short stature, the majority of the population (87.5%) identified familial (hereditary) short stature as a cause for short stature. This high percentage might be attributed to the visible transmission of height patterns within families, making hereditary causes more intuitive and familiar to the general public than other medical causes.

This aligns with the findings of a study conducted at the Pediatric Endocrine Clinic of King Khalid University Hospital (KKUH), King Saud University (KSU) in Riyadh, which reviewed the patterns of short stature in 110 referred patients and concluded that the most common cause of short stature was familial in nature [10]. These results are consistent with the predominance of physiological short stature types such as familial short stature and constitutional delay, as found in local studies. In contrast, pathological causes, such as endocrine disorders or malnutrition, were less common but more clinically significant. A recent cross-sectional study from Taif, Saudi Arabia, found that 40.8% of children with short stature had familial short stature, 24.2% had malnutrition, and 9.7% had growth hormone deficiency, followed by 7.6% with hypothyroidism [11].

Moreover, more than half of the participants (58.6%) recognized hormonal imbalances, such as growth hormone deficiency or hypothyroidism, as contributing factors to short stature. This finding may indicate relatively higher exposure in Hail to endocrine-related information, perhaps due to local clinical experiences or social media dissemination of hormone-related conditions.

This percentage is considerably higher than that reported in a national study involving participants from all the regions across Saudi Arabia, with nearly half (45.3%) from the central region, where only 26.2% identified endocrine disorders as a cause of short stature [12]. Such regional differences could reflect varying levels of access to specialized pediatric care or differences in community health priorities.

Malnutrition was identified by 46.4% of our participants as a cause of short stature, which reflects a moderate level of understanding. However, this may indicate that the public tends to underestimate the impact of nutritional status on child growth, possibly due to the assumption that malnutrition is not a prevalent issue in the region.

This is notably lower than the findings in the comparative national study [12], where 70.9% and 60.9% of participants identified poor food quality and reduced meal frequency, respectively, as contributing factors. This difference may be attributed to regional disparities in health education and public awareness. 

It is possible that in the Hail region, public health campaigns and nutritional education are less emphasized compared to larger metropolitan areas. This may suggest a need for more regionally tailored public health initiatives in the Hail region to strengthen awareness of nutritional causes of growth disorders.

Awareness of other contributing factors in our study was generally limited. Only 29.6% of the participants recognized staying up late as a potential contributor, 27.8% identified genetic syndromes, and just 6.1% were aware of psychological causes. This limited awareness may reflect cultural or educational gaps related to the broader determinants of growth, particularly psychosocial and environmental influences.

In contrast, the national study [12] reported that 47.3% of participants acknowledged child abuse and neglect as potential causes of short stature. This contrast may indicate that in the Hail region, topics related to mental health and child welfare receive less emphasis in public discourse and health education, which could contribute to the lower recognition of psychosocial factors as causes of short stature. In comparison, the higher awareness observed in the national sample suggests broader exposure to these issues at the national level. 

Overall, while participants demonstrated relatively good knowledge of familial and hormonal causes, there was limited recognition of less obvious but clinically significant contributors such as nutritional deficiencies, chronic diseases, and psychosocial factors. This gap in understanding may delay appropriate referrals and hinder the identification and management of modifiable underlying causes during pediatric evaluations, highlighting the need for targeted health education campaigns in the Hail region to enhance community awareness of the multifactorial nature of short stature in children.

The majority of participants (82.5%) believed that intervention is needed before puberty if short stature is noticed, reflecting a positive level of awareness among the participants regarding the critical timing for addressing short stature. 

This awareness may reflect exposure among the local population to the idea that growth slows after puberty, possibly through school health education or informal sources such as social media.

Most participants (61.1%) recognized that short stature is not always pathological and can sometimes be a normal variant. This awareness may help reduce unnecessary concern or medicalization in cases of normal variation, such as constitutional growth delay. However, the remaining proportion reflects potential confusion between normal and abnormal growth, indicating a need for further education to distinguish normal variants from underlying pathological conditions that require timely intervention.

Regarding the choice of healthcare provider, 65.4% of participants recognized the need to consult a pediatric endocrinologist if short stature was observed, whereas 64.7% believed that a general pediatrician was the appropriate specialist, and only 8.6% selected internal medicine.

This overlap in provider selection may reflect uncertainty or lack of awareness regarding medical subspecialties and may suggest that the public might not fully understand referral pathways in pediatric care. 

A similar pattern was observed in a study conducted in the Qassim region, where 52.0% of participants believed a pediatrician was the most qualified specialist to consult, while 34.5% selected endocrinologists, and only 2.3% chose internal medicine [8]. This confusion may reflect limited public exposure to the pediatric endocrine specialty in the region, highlighting the need for targeted educational initiatives to clarify the distinct yet collaborative roles of general pediatricians and pediatric endocrinologists in the diagnosis and management of growth disorders. Improving this understanding could enhance the efficiency of referral systems and reduce delays in appropriate care.

Regarding treatment, 60.3% of the study population believed that therapeutic interventions, particularly hormone replacement therapy, were the primary approach for managing short stature. Meanwhile, 38.5% reported that both therapeutic and surgical interventions could be used, and only 1.2% considered surgical intervention as the sole treatment option. 

The relatively high expectation for surgical treatment among participants may be influenced by media portrayals of height-enhancing surgeries and a lack of clear understanding that surgical intervention is generally reserved for rare, severe skeletal disorders. This overestimation highlights the need for educational efforts to clarify the limited role of surgery in managing short stature and to promote realistic expectations tailored to the specific diagnosis of each child. 

In comparison, the same study from the Qassim region also reported that 68.0% of respondents believed that both therapeutic and non-therapeutic interventions could be appropriate, while 26.0% selected therapeutic interventions alone, and only 6.0% considered non-therapeutic approaches exclusively [8]. Collectively, these findings underscore the importance of enhancing public education on the timing, appropriate specialists, and nature of treatment options for short stature in children to promote accurate health-seeking behavior and informed decision-making.

Our study revealed that the majority of participants (74.8%) had poor knowledge of short stature in children, while only 25.2% demonstrated good knowledge. This underscores the widespread lack of awareness surrounding the condition and may indicate that current health education efforts in the region are insufficient or not effectively reaching the target population.

This finding is consistent with a descriptive study conducted in Egypt, which demonstrated that 75% of mothers had poor knowledge of short stature, and 87.5% of mothers of children with short stature believed that their children were of normal height [13]. These findings emphasize the need for culturally appropriate and accessible educational programs to improve knowledge and early recognition of short stature among caregivers and the general public.

Interestingly, despite the generally poor knowledge regarding short stature among the study participants, the majority (85.3%) reported that they would seek medical advice if their children were significantly shorter than their peers. A small percentage (12.8%) were unsure, and an even smaller proportion (1.9%) indicated that they would not seek medical attention. This finding suggests that participants may rely on general health awareness, social norms, or personal concern rather than specific medical knowledge when deciding to seek professional care. Such a tendency among participants to seek medical advice represents an opportunity to bridge the gap between intention and informed action through targeted educational interventions.

The findings of this study revealed that participants who were young (18-25 years old), educated, unmarried, students, and non-parents were significantly more likely to have better knowledge.

This association may be explained by increased exposure to digital health content and structured health education, especially through universities and social media platforms, which are more commonly accessed by younger and more educated individuals. These findings suggest that certain sociodemographic factors can influence awareness and understanding of short stature. In contrast, older or married individuals may rely more heavily on traditional knowledge sources or personal experiences, which may not always provide accurate or up-to-date information. 

These results contrast with those of the study conducted in the Qassim region, which reported that sociodemographic factors such as being married, having children, and having a family history of short stature were associated with better knowledge [8]. This difference may reflect variations in the study populations, levels of health education, or regional exposure to healthcare information. It may also suggest differing patterns of reliance on lived experience versus formal education as sources of health knowledge.

However, other sociodemographic factors, such as sex, nationality, and place of residence, did not show significant associations with knowledge levels in our study.

The cross-sectional design of this study limits its ability to establish causal relationships between the examined variables, as it captures data at a single point in time rather than observing changes over time. In addition, the reliance on self-reported data introduces recall and social desirability biases, which may affect response accuracy. The use of non-probability convenience sampling also limits the generalizability of the findings. The questionnaire was adapted from a published study and reviewed by a pediatric consultant to ensure face validity; however, no formal statistical validation was performed. This study also did not include multivariate analysis to control for potential confounding factors or to assess the independent associations between sociodemographic variables and knowledge levels. Future studies should consider using logistic regression or other multivariate techniques to provide more comprehensive insights. These limitations should be considered when interpreting the findings, as they may affect the generalizability of the results to broader populations.

The strengths of this study include a relatively large sample size and a focus on an understudied region, which fills a critical knowledge gap.

## Conclusions

In conclusion, this study found that the majority of participants from the Hail region demonstrated poor knowledge of pediatric short stature, with only a small proportion achieving a good knowledge score based on predefined criteria. Younger age, higher education level, unmarried status, and being a non-parent were significantly associated with better knowledge. These findings indicate gaps in awareness that are particularly evident among older adults, parents, and individuals with lower educational attainment.

Therefore, tailored educational interventions focusing on these subgroups are warranted to improve understanding of pediatric short stature, promote early recognition, and encourage timely healthcare seeking in the region.

## Appendices

Research questionnaire

Arabic Version

الموافقة المستنيرة  

نحن فريق بحثي من كلية الطب-جامعة حائل يهدف إلى معرفة مدى ادراك ووعي السكان في منطقة حائل - المملكة العربية السعودية تجاه قصر القامة لدى الأطفال.  

يرجى قراءة المعلومات التالية بعناية, سيتم التعامل مع جميع المعلومات التي تقدمها بسرية تامة ولن يتم الكشف عن هوية أي مشارك/ مشاركة . 

مشاركتك طوعية تماماً ونقدرها كثيراً. 

１- العمر: 

·      ١٨-٢٥

·      ٢٦-٣٥

·      ٣٦-٤٠

·      ٤١-٥٠

·      + ٥٠

２- الجنس: 

·      أنثى

·      ذكر 

３- الجنسية: 

·      سعودي 

·      غير سعودي

４- مكان الإقامة: 

·      في حائل 

·      في القرى المجاورة 

５- المستوى التعليمي: 

·      أمي

·      ابتدائي 

·      متوسط 

·      ثانوي

·      جامعي

·      دراسات عليا

６- الحالة الاجتماعية: 

·      أعزب/عزباء

·      متزوج/ة

·      مطلق/ة

·      أرمل/ة

７- أين تعمل؟

·      لا أعمل

·      في قطاع الصحة 

·      في قطاع التعليم

·      أخرى

８- هل لديك أطفال؟ 

·      نعم

·      لا

·      أرعى أطفال لكنهم ليسوا أطفالي

９-   متى تبدأ ملاحظة أن الطفل قصير القامة (تعريف قصر القامه عند الأطفال)؟ (يمكنك اختيار أكثر من إجابة)

·      عندما يلاحظ أن الطفل قصير مقارنة مع اخوته أو أقرانه من نفس العمر والجنس 

·      تأخر في تغيير مقاسات الطفل في الملابس واستمرار لبسها لوقت أطول 

·      وقوع طول الطفل بالانحراف المعياري تحت ٢ أو أقل من ٣٪  في منحنيات النمو بالرسوم البيانية للأطفال المتوافرة في العيادات 

１０-             باعتقادك متى يجب زيارة الطبيب في حال لاحظت قصر القامة على طفلك؟

·      قبل بلوغ الطفل

·      بعد بلوغ الطفل 

１１-             ماهي أسباب قصر القامة لدى الأطفال؟ (يمكنك اختيار أكثر من إجابة) 

·      عائلية وراثية (قصر القامة الوراثي) 

·      تأخر في سرعة النمو

·      أمراض هرمونية (نقص هرمون النمو أو نقص هرمون الغدة الدرقية)

·      أمراض مزمنة (أمراض القلب، أمراض الكبد، أمراض الكلى ،مشاكل امتصاص في الجهاز الهضمي، الربو) 

·      أمراض نقص المناعة 

·      أمراض جينية (متلازمات) 

·      مشاكل نفسية 

·      السهر 

·      سوء التغذية 

·      أخرى 

１２-             هل يمكن أن يعتبر قصر القامة شكل طبيعي من أنماط النمو المختلفة؟ 

·      نعم في بعض الحالات 

·      لا، إطلاقًا 

·      لا أعلم

１３-             من هم أخصائيو الرعاية الصحية الذين يجب استشارتهم إذا كان الطفل يعاني من قصر القامة؟ (يمكنك اختيار أكثر من إجابة) 

·      طبيب أطفال عام 

·      طبيب الباطنة

·      طبيب غدد صماء - أطفال

·      أخرى 

１４-             ما نوع التدخلات التي يمكن استخدامها لعلاج قصر القامة؟

·      تدخل دوائي (علاج هرموني تعويضي)

·      تدخل جراحي

·      كلاهما

１５-             هل يوجد شخص في عائلتك يعاني من  قصر القامة؟

·      نعم

·      لا 

１６-             اذا كان جوابك نعم، متى تم اتخاذ إجراءات لحل المشكله؟

·      قبل البلوغ

·      بعد البلوغ

·      لم يتعرض لأي تدخل طبي

１７-             هل ستفكر في طلب المشورة الطبية إذا كان طفلك أقصر بكثير من أقرانه؟

·      نعم 

·      ربما 

·      لا

English Version

Informed consent  

We’re a medical research group from Hail University working to assess the Knowledge and Perception of The Population in Hail Region - Kingdom of Saudi Arabia Towards Pediatrics Short Stature in Children.  

Please read the following instructions carefully, all information given will hold confidentiality and no personal information will be shared.  Your participation is fully voluntarily and appreciated. 

1-     Age: 

·      18-25

·      26-35

·      36-40

·      41-50

·      + 50

2-     Sex: 

·      Female 

·      Male 

3-     Nationality:

·      Saudi

·      Non-saudi

4-     Place of residence: 

·      Hail

·      Villages near Hail

5-     Educational Level:

·      Illiterate

·      Elementary school 

·      Middle school 

·      High school 

·      University

·      Postgraduate studies 

6-     Marital Status: 

·      Single 

·      Married 

·      Divorced 

·      Widowed 

7-     Where do you work? 

·      Unemployed

·      In the health sector 

·      In the education sector 

·      Other 

8-     Do you have children?

·      Yes

·      No 

·      I foster children who are not my own 

9-     When do you begin to notice that the child has short stature (definition of short stature in children)? (You can choose more than one answer)

·      When you notice that the child is short compared to his siblings or peers of the same age and gender. 

·      Delay in changing the child’s clothing sizes and continuing to wear them for a longer time. 

·      When the child’s height is below 2 standard deviation or less than 3% in the growth curves in the charts for children available in clinics. 

10-  When do you think you should visit a doctor if you noticed that your child has short stature?

·      Before puberty 

·      After puberty 

11-  What are the causes of short stature in children? (You can choose more than one answer)

·      Familial (hereditary short stature)

·      Constitutional Delay

·      Hormonal causes (Growth Hormone Deficiency or Hypothyroidism)

·      Chronic diseases (Heart diseases, Liver diseases, Kidney diseases, Malabsorption, Asthma)

·      Immunodeficiency diseases

·      Genetic diseases (Syndromes)

·      Psychological causes

·      Staying up late at night

·      Malnutrition

·      Other

12-  Can short stature be considered a normal form of growth disorders?

·      Yes in some cases

·      No, never

·      I don’t know 

13-  Which healthcare professionals should be consulted if a child has short stature? (You can choose more than one answer)

·      General Pediatrician

·      Internal Medicine

·      Pediatric Endocrinologist

·      Other 

14-  What type of interventions can be used to treat short stature?

·      Therapeutic interventions (Hormone Replacement)

·      Surgical interventions

·      Both 

15-  Do you have a family history of short stature?

·      Yes

·      No

16-  If yes, when was the medical intervention initiated?

·      Before puberty 

·      After puberty 

·      They didn’t seek any medical intervention

17-  Would you consider seeking medical advice if your child was significantly shorter than their peers?

·      Yes

·      Maybe 

·      No 
